# The Cost-Effectiveness of Mobile Health (mHealth) Interventions for Older Adults: Systematic Review

**DOI:** 10.3390/ijerph17155290

**Published:** 2020-07-22

**Authors:** Zartashia Ghani, Johan Jarl, Johan Sanmartin Berglund, Martin Andersson, Peter Anderberg

**Affiliations:** 1 Department of Health, Blekinge Institute of Technology, SE-371 79 Karlskrona, Sweden; johan.sanmartin.berglund@bth.se (J.S.B.); peter.anderberg@bth.se (P.A.); 2 Health Economics Unit, Department of Clinical Sciences, Lund University, SE-221 00 Lund, Sweden; johan.jarl@med.lu.se; 3 Department of Industrial Economics, Blekinge Institute of Technology, SE-371 79 Karlskrona, Sweden; martin.andersson@bth.se

**Keywords:** aged, cost-benefit analysis, economic evaluation, gerontechnology, telemedicine

## Abstract

The objective of this study was to critically assess and review empirical evidence on the cost-effectiveness of Mobile Health (mHealth) interventions for older adults. We systematically searched databases such as Pubmed, Scopus, and Cumulative Index of Nursing and Allied Literature (CINAHL) for peer-reviewed economic evaluations published in English from 2007 to 2018. We extracted data on methods and empirical evidence (costs, effects, incremental cost-effectiveness ratio) and assessed if this evidence supported the reported findings in terms of cost-effectiveness. The consolidated health economic evaluation reporting standards (CHEERS) checklist was used to assess the reporting quality of the included studies. Eleven studies were identified and categorized into two groups: complex smartphone communication and simple text-based communication. Substantial heterogeneity among the studies in terms of methodological approaches and types of intervention was observed. The cost-effectiveness of complex smartphone communication interventions cannot be judged due to lack of information. Limited evidence of cost-effectiveness was found for interventions related to simple text-based communications. Comprehensive economic evaluation studies are warranted to assess the cost-effectiveness of mHealth interventions designed for older adults.

## 1. Introduction

Gerontechnology is an interdisciplinary research area in which disciplines such as healthcare, industrial design, and architecture interact in using advancements in technology to tackle challenges posed by an aging society [1]. One aspect of gerontechnology and the focus of the present study is mobile health (mHealth). This is a means to provide remote assistance to individuals with chronic health conditions as well as to reduce strain on healthcare budgets [2]. It includes the use of mobile phones and other wireless devices to support the healthcare delivery process and disseminate information [3].

Interest in policy actions aimed to support aging in place with the help of technical assistance is substantial and calls for economic evaluation (EE) of mHealth interventions. EEs are an integral part of health technology assessments (HTA) and compare the costs and benefits of intervention with two or more alternatives to assess forgone benefits (opportunity cost) of alternative treatments. EEs thus constitute cost-effectiveness analyses and are essential for informed decisions about the allocation of healthcare resources efficiently and equitably.

Prior systematic reviews evaluated the quality of reporting of EEs of mHealth interventions among all age groups [4] and of eHealth interventions among older adults [5]. High quality of reporting is expected to result in high quality of study, although this does not necessarily hold true in all cases. Therefore, it is also important to critically assess the results of included studies to facilitate the reader’s interpretation of the findings and to determine the usefulness of the EEs for informed decision-making. Neither of the prior systematic reviews focused on this aspect. Therefore, besides the quality of reporting, the present study aimed to assess if the findings of the included studies are enough to establish the cost-effectiveness of mHealth use for elderly care. Thus, we intend to gather information on general characteristics, results of EEs of mHealth interventions, and to appraise these results critically.

## 2. Materials and Methods

### 2.1. Protocol and Registration

The review was registered with PROSPERO International Prospective Register of Systematic reviews on July 24, 2018; registration number CRD42018100042.

### 2.2. Search Strategy

Although there is no consensus on how many databases should be searched for writing a systematic literature review (SLR), the recommendation in the literature is to search at least two bibliographic databases [6]. We systematically searched three large databases: *Pubmed*, *Scopus, and* Cumulative Index of Nursing and Allied Health Literature (CINAHL) to identify relevant articles published in peer-reviewed journals between 2007–2018. Since most relevant articles appear on the first few pages of *Google Scholar*, we assessed the first 50 articles on May 29, 2019 to complement our main search. We also searched for additional articles in the reference lists of selected studies and in citations made on included studies. Keywords (Table S1) used, and search strategies are reported in Supplementary File 2.

### 2.3. Inclusion and Exclusion Criteria

The criteria for article selection were: (1) interventions that relate to the delivery of healthcare and/or dissemination of healthcare information using portable devices having internet connection and/or software applications [7,8]; (2) older adults aged 55 years or above; (3) full economic evaluation such as cost-benefit analysis (CBA), cost-utility analysis (CUA) or/and cost-effectiveness analysis (CEA) as well as cost-minimization analysis (CMA); and (4) published in English in peer-reviewed journals.

The criteria for article exclusion were: (1) studies related to cost analysis such as cost of illness; (2) systematic literature reviews, commentaries (letter to the editors, editorials), abstracts, etc.; and (3) studies related to telephone interview or survey or questionnaire or recruitment of trial participants by telephone. Studies related to telephone counseling, non-automated follow-up calls, or required health professionals’ involvement were also excluded.

### 2.4. Study Selection Process and Data Extraction

After removing the duplicate studies, two reviewers independently screened the title and abstract of all records to identify relevant studies based on the inclusion and exclusion criteria. After this, the full text of selected articles was assessed against the inclusion criteria and reason for exclusion of studies was recorded at this stage.

Data on the results of the selected studies (empirical evidence) and how those results were derived (methodology) was collected. For instance, we collected data on costs, health outcome, and incremental cost-effectiveness ratio (ICER, if reported) from selected studies. We also collected data on health outcomes from selected studies to identify whether health outcomes were measured as utility index or as other outcomes, such as depression-free days (DFD), unified Parkinson’s disease rating scale (UPDRS), etc. In cases of missing information, authors of selected studies were contacted through email (maximum of three email attempts).

Furthermore, we reported the authors’ conclusions about the cost-effectiveness of the intervention and if the reported information supported the authors’ findings and conclusions. When possible, we also analyzed cost-effectiveness acceptability curves (CEAC), which help to establish the cost-effectiveness of an intervention in the absence of reporting on significance level (e.g., 95% CI). A range exists of values of willingness to pay (WTP) for quality adjusted life years (QALY) to determine the cost-effectiveness of intervention. In the UK, for example, a threshold range of £20,000–£30,000 is used while $50,000 per QALY gained is used in the USA. In this SLR, we used a threshold of USD 50,000/QALY [9] for determining the cost-effectiveness of included studies.

### 2.5. Assessment of Quality of Reporting

The consolidated health economic evaluation reporting standards (CHEERS) [10] guideline was used to assess the quality of reporting in the studies included in the review. The CHEERS guideline aims to harmonize the information presented in studies and to improve the quality of EEs. The CHEERS checklist covers 24 items belonging to six categories (title and abstract, introduction, methods, results, discussion, and other). For each item, we classified each study into three mutually exclusive categories: ‘Yes’ (reported in full), ‘No’ (not reported), and ‘Not Applicable’. Further, we assigned a score of one if an item belonged to either ‘Yes’ or ‘Not applicable’ categories and zero otherwise. Thus, the maximum score for full reporting was 24 for each article. We were prompted to critically appraise the included studies in the discussion section using Drummond’s checklists [11].

## 3. Results

### 3.1. Search Results

We retrieved 1853 potentially relevant studies from three databases (Pubmed, Scopus and CINAHL) and subsequently removed 712 duplicate studies. Two review team members independently screened the title and the abstract of the remaining 1141 records based on inclusion criteria, leaving 54 potential articles for the full-text reading. After reading the full text of these 54 articles, 18 articles were excluded for the following reasons: technology used by experts (six studies), not home-care (two studies), PhD thesis (one study), not focusing on older adults (three studies), not full EE (two studies), implanted ICD/pacemaker (three studies) and obstructive sleep apnea (one study). Further, eight more studies were identified through reference and citation searches.

**Figure 1:**
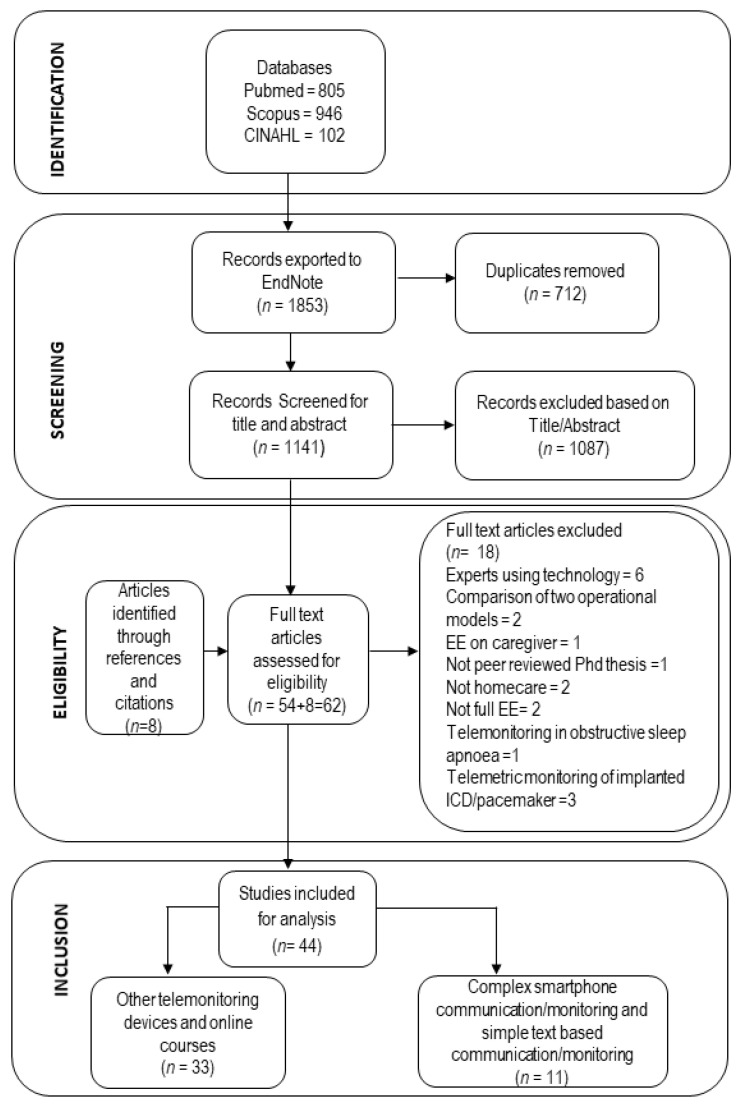
PRISMA flow chart of study selection process.

Since monitoring devices used in mHealth interventions are diverse, we have categorized all the interventions into two closely related intervention groups: (1) “complex smartphone communication” and “simple text-based communication”; (2) monitoring devices other than smartphone and website/online courses. Thus, in total 44 studies fulfilled the inclusion and exclusion criteria; eleven of those studies belonged to intervention group 1 and were analyzed in the present review. Figure 1 shows the preferred reporting items for systematic reviews and the meta-analysis (PRISMA) flow chart for the study selection procedure [12]. The remaining 33 studies belonged to intervention group 2 and will be analyzed in the subsequent paper.

### 3.2. Overview of Included Studies

Tables S2–S5 (Supplementary File 1) presents detailed characteristics of the studies included in the review. Most of the studies were conducted in Anglosphere countries. One of the 11 studies was conducted in 2007 [13] and the remaining studies between 2013–2017. These studies included individuals from 57–75 years of age, with the majority in their sixties. We found four studies related to heart disease [14,15,16,17], two related to chronic obstructive pulmonary disease (COPD) [18,19], three related to diabetes [13,20,21] and two studies related to Parkinson’s disease (PD) [22] and cancer [23].

The perspective of an economic evaluation is important as it determines which types of costs and health benefits are to be included in the analysis. Most studies used a healthcare perspective [15,16,18,19,21]. Four studies did not report their perspective, though it was possible to infer their perspective based on the contents of the study. Katalenich et al. [20] followed a healthcare perspective by including only intervention costs, physician visit costs, and cost for follow-up calls. Cubo et al. [22] used a limited societal perspective by focusing only on direct medical and direct non-medical costs. Maddison et al. [17] used a limited healthcare perspective by only including costs associated with the delivery and implementation of the intervention, and Barnett et al. [13] followed the payer’s perspective by including direct costs to the Department of Veterans Affairs.

### 3.3. Results of Included Studies

#### 3.3.1. Complex Smartphone Communication

Cubo et al. [22] performed CUA and CEA of a one-year intervention designed for Spanish patients with advanced PD. Patients were provided with a tablet software app for home-based motor monitoring (HBMM) plus regular in-office visits, compared to office-based management (OBM) alone. This study used both QALY and disease-specific (improvement in UPDRS I, II, III, IV motor ratings) outcome measures to calculate ICER. ICERs for UPDRS part II, III, IV and UPDRS-total range from €126.72/UPDRS to €701.31/UPDRS.

We found two studies evaluating touch-screen interface/tablet with two apps for COPD patients in-home care settings. Stoddart et al. [18] performed a CUA where COPD patients were randomized into touch-screen telemonitoring with usual care compared to usual care alone in the UK for 12 months. From the national health service (NHS) perspective, the ICER of telemonitoring compared to usual care was found to be £137,277/QALY (base case scenario). However, exclusion of hospital admissions for other reasons than COPD and exclusion of telemonitoring equipment, set up and broadband costs reduced the estimated ICERs to £67,797/QALY and £46,828/QALY, respectively [18].

In the other study, in Denmark, Udsen et al. [19] performed a CEA for COPD care in a home-care setting. In the intervention group, patients received standard tablets with two apps and peripherals (fingertip pulse Oximeter, a digital blood pressure monitor, and a scale) in addition to usual care. For 12 months, the base-case ICER of the intervention group compared to usual care was €55,327/QALY from the healthcare and social sector perspective. Three sensitivity analyses were performed by including all-cause hospital contacts (ICER: €44,301/QALY), reduced procurement prices (ICER: €46,931/QALY), and reduced monitoring time from five to two minutes (ICER: €39,854/QALY) followed by a fourth sensitivity analysis which included all of these scenarios (ICER: €21,068/QALY).

Whittaker et al. [14] evaluated the costs and benefits of a home telehealth-based cardiac rehabilitation program in comparison with standard hospital-based programs for cardiovascular disease (CVD) patients residing in Australia. The intervention was carried out both from the provider’s and participant’s perspectives over six months. The intervention consisted of a smartphone, Wellness Diary, and a Wellness web portal, daily motivational text messages, and transfer of patient’s measurements via smartphone, whereas standard care included a six-week hospital-based outpatient cardiac rehabilitation program, including gym sessions. Based on equal clinical outcomes in both intervention and control groups, the authors confined their analysis to a comparison of costs. The intervention group had lower cost (AUD1633 per patient) compared to the usual care group (AUD1845 per patient) from the provider’s perspective, and the intervention group also had lower travel expenses (AUD80) compared to usual care group (AUD400) from the participant’s perspective.

Gordon et al. [21] used a Markov model for automated telephone-linked care (TLC) compared with usual care for patients with Type 2 diabetes mellitus in Australia. The intervention consisted of a handbook, blood glucose meter, mobile phone, Bluetooth device and box of test strips. This five-year Markov model with a cycle length of six months had four health states: sub-optimal glycemic control, average glycemic control, optimal glycemic control, and all-cause death. The effectiveness data came from a TLC diabetes intervention on 120 patients for 24 weeks. In the base model, TLC costs were reduced by £683 and 0.004 QALYs were gained. Although base-case results remained against most of the one-way sensitivity analyses of different parameters (e.g., discount rate, starting age, etc.), these results were sensitive to model duration, utilities, and medication costs.

#### 3.3.2. Simple Text-Based Communication

In the US, automated symptom monitoring was evaluated as a part of a centralized telecare management intervention in comparison to usual care from a payer’s perspective for 12 months [23]. Of the 405 patients, a sub-group analysis was performed on 154 depressed patients using DFD as outcome measures. Sensitivity analyses for ICERs were reported by computing QALYs in three different ways: (i) from DFDs, (ii) six-dimensional health state short form (SF-6D), and (iii) modified EuroQol five-dimension scale (EQ-5D) survey. The ICER/DFD for complete and imputed follow-ups was estimated at US$27/DFD, whereas ICERs derived from EQ-5D and SF-6D were estimated at US$10,826/QALY and US$73,287/QALY, respectively [23].

Three of the included economic evaluations focused on patients with cardiovascular diseases [15,16,17]. From a healthcare perspective, Burn et al. [15] used a Markov model for motivational text messages (TEXT ME), in addition to usual care, compared with usual care alone for patients with documented coronary heart disease (CHD) in Australia. The lifetime Markov model with a cycle length of six months had six health states: a history of CHD, myocardial infarction (MI), stroke, history of MI, history of stroke and death. The effectiveness data came from a randomized controlled trial of TEXT ME on 710 patients for 6 months. Under base-case assumptions, costs were reduced by AUS$ 10.56 million (95% CI: −14.4 to −7.4), and 1143 QALYs (95% CI: 717 to 1667) were gained. Results from sensitivity analyses for four different scenarios showed that TEXT ME remained dominant (less expensive and more effective) for all these scenarios and reported an ICER of AUS$ 6123/QALY for simultaneous sensitivity analysis of all four scenarios.

Maddison et al. [17] performed both CEA and CUA of a mobile-phone delivered intervention (HEART—Heart, exercise, and remote technologies) alongside usual care, compared with usual care alone, for patients with ischemic heart disease (IHD) in New Zealand. This 24-week intervention included personalized automated text messages and a website with video messages. The effectiveness measures used in this analysis are QALY and metabolic equivalent (MET)-hour of walking per week and leisure activity per week. The HEART intervention resulted in an ICER of NZD 28,768/QALY and ICERs of NZD 48 and NZD 74 per MET-hour of walking per week and leisure activity per week, respectively.

In one CEA, two different intervention combinations were compared to standard care in Canada. The interventions were health lines (HL) plus standard care, and health lines plus monitoring (HL + M) plus standard care. In health lines, nurses could be contacted for advice on daily disease management. Monitoring consisted of home monitoring devices accompanied by a computerized call scheduled to remind patients to record their weight and blood pressure data. The analysis was performed from the perspective of the healthcare system on patients with congestive heart failure over a period of 12 months. Both HL and HLM were less costly and more effective compared to standard care, thereby both interventions dominated standard care. Further, HL improved QALYs by 0.04 (statistically significant) and led to increased costs of CAD 119 (statistically insignificant) per patient versus HLM. The ICER for HL versus HLM was CAD 2975/QALY and remained robust after sensitivity analysis for uncertainty in costs and outcomes of the intervention [16].

In two studies from the US, EEs of remote monitoring for people with diabetes were conducted. Katalenich et al. [20] performed CEA and CMA for the automated diabetes remote monitoring and management system (DRMS) intervention, including automated voice phone calls or text messages to remind patients to test their blood glucose, reporting results via an automated system and automated advice from DRMS on insulin adjustment, compared to usual care, for six months. QALYs were estimated based on a prediction model of clinical characteristics using age, sex, blood pressure, low-density lipoprotein, and HbA1c value. The intervention had a lower cost (statistically significant) and improved QALY score (statistically and clinically insignificant) compared to usual care.

In the other study, Barnett et al. [13] performed a CUA to compare the care coordination/home telehealth (CCHT) program before and after the introduction. In this program, 370 diabetes patients entered answers to questions about their diabetes symptoms using a messaging device daily for 12 months. The same cohort was also observed before the introduction of the CCHT program for 12 months. The evaluation reported an overall mean ICER of US$60,941/QALY for the full sample. The result of sub-analyses (logistic regressions) showed that the intervention was more cost-effective for married patients and patients with a history of myocardial infarction and hemiplegia or paraplegia.

## 4. Discussion

This SLR assessed available empirical evidence on the cost-effectiveness of mHealth interventions for older adults. The results of different cost-effectiveness analyses are not directly comparable due to their design.

Table 1 presents the results on the cost-effectiveness of the included studies as reported by their authors, along with our assessment of the reported information. Reporting of confidence intervals (CIs) is important to establish the differences between costs and/or outcomes between the intervention and the comparator before computing the ICER. In the absence of CIs (or a corresponding test), we follow a conservative approach by assessing the cost-effectiveness as “unknown due to lack of information”.

The concept of cost-effectiveness is subjective as it depends on the decision maker’s WTP for a particular outcome. Therefore, authors of EEs should make an explicit valuation of outcomes to determine the cost-effectiveness of an intervention. The acceptable WTP threshold to establish the cost-effectiveness of intervention differs in the literature. For instance, in the UK, a threshold range of £20,000–£30,000/QALY gained is used [24], while American [9] and Australian [25] studies used the amount of 50,000/QALY gained in their respective currencies. There have also been arguments against the use of cost-effectiveness thresholds. Instead, the emphasis has been put on context-specific decision-making by taking into account the targeted patient group, legislation, etc. [26]. While it is easier to establish cost-effectiveness for QALY against the well-established threshold levels, the use of disease-specific instruments implies a need for clear discussion and motivation to establish an appropriate valuation. This is crucial in a decision-making context because it is a prerequisite to be able to assess the cost-effectiveness of the outcomes under study. However, an intervention will be preferable irrespective of the valuation of the outcome measure if it improves the outcome and is less expensive compared to the comparator (scenario 2 and 4 in Table 2), and vice versa. This is also true in cases where cost remains indifferent between treatment arms (intervention and control group) and a decision must be made based on increase/decrease in outcome, and vice versa (scenario 3, 6, 7 and 8 in Table 2). In this discussion, we consider all clearly preferred alternatives as cost-effective.

Despite the absence of a precise threshold for QALY, policymakers and researchers still use and rely on threshold values for QALY to compare the cost-effectiveness of different interventions. However, there is no consensus on valuation for other effectiveness measures such as UPDRS, MET hour of walking, DFDs etc. (Table 1). In cases like these, an excellent way to establish a societal valuation of the outcomes is to include prior studies related to the implementation of the same type of outcomes, potentially within the same disease area. None of the included studies made any rigorous justifications of the societal valuation of used outcomes other than QALY. In Cubo et al. [22], for instance, the authors stated HBMM cost-effective in terms of improvement in functional status (UPDRS) with an ICER of €126.72 per unit of UPDRS. However, it was not established that the societal valuation of one additional point in UPDRS is above €126.72. This also applies to other effectiveness measures such as MET-hour of walking and leisure activity, and DFD used in other identified studies [17,23]. Lack of sufficient evidence or threshold level for outcome measures other than QALY preclude our judgment to decide their cost-effectiveness [14,22], and we refer to this evidence as “unknown due to no agreed cost-effective threshold value” (Table 1).

Our judgment about cost-effectiveness is in line with the reported conclusions in five studies [15,16,18,19,20]. In three studies [16,18,19], mHealth interventions were not significantly different from their comparators in terms of costs and benefits whereas, in the remaining two studies, the intervention was significantly better and cheaper, [15] and costs were significantly lower for the intervention group [20].

Moreover, some studies deemed intervention group as cost-effective although we assessed their results as “unknown due to lack of information” because information on differences between either costs or/and benefits were not reported [13,14,17,22,23]. Gordon et al [21] reported the intervention group as dominant even though they did not find significant differences in costs and outcomes between intervention groups. In our assessment, an intervention cannot be stated to be better than the alternative if it is not effective in either improving the outcome or reducing the costs. This means that an ICER based on insignificant differences is not reliable. Therefore, we contradicted the author’s finding and reported our assessment as ‘not cost-effective’.

CIs were not reported in many studies [13,14,17,22,23]. Due to the diverse nature of costs and effects, it is not easy to find significant differences between the two comparators. However, it is still important to establish whether there are differences between the alternatives in an economic evaluation in terms of costs and effects before considering cost-effectiveness.

As shown in Table 1, few studies included CEAC to deal with uncertainties around costs and effects [16,18,19]. We urge authors to include CEAC in EEs as it is not only a good practice but it also serves as an alternative to producing CIs around ICER. However, there is no consensus among researchers on which value a CEAC should be referred to consider intervention as cost-effective. For ease of comparison, we consider intervention as being “cost-effective” if the intervention was 80% likely to be cost-effective at the threshold of USD 50,000/QALY. It is also essential to recognize that the implementation of an intervention should not only be based on findings from CEAC, and findings should be considered in light of the uncertainty around the estimated cost-effectiveness ratio [27]. For example, Maddison et al. [17] suggested that the HEART intervention was not cost-effective with an ICER of NZ$28,768 (€15,247) per QALY, as its ICER was not under the threshold of NZ$20,000/QALY. Although no significant differences were observed in QALY or costs between intervention and control groups, Maddison et al. [17] showed 90% probability of this intervention being cost-effective at WTP of NZ$20,000/QALY. Therefore, we considered this intervention cost-effective based on the threshold level of USD 50000/QALY used in the present study.

Based on our assessment of the results of the included studies, evidence on the overall cost-effectiveness of the EEs in mHealth interventions is limited. In light of available evidence, we encourage authors to refrain from reporting their results as cost-effective in favor of mHealth interventions without presenting substantial evidence to back up their claims. We strongly urge researchers to use the term “cost-effective” with caution to avoid giving the wrong impression. For example, Katalenich et al. [20] used the term cost-effectiveness while only discussing the costs incurred by intervention and control groups. The same applied to a study by Whittaker et al [14]. 

We further observed that Cubo et al. [22] tended to highlight results for UPDRS (II III IV) as they showed improvement in the intervention group. One study also tried to calculate differences in outcome (SF-6D) based on a previous study conducted on Australian patients when they did not find a statistically significant difference in SF-6D score within their study [21]. Interestingly, Whittaker et al. [14] also reported savings, in terms of re-admission, in CBA while claiming equal clinical outcomes in the same study. Savings in healthcare costs in the presence of equal clinical outcomes still made the results questionable. Further, the authors did not report data related to equal clinical outcomes and did not discuss the reasons behind their claim of equal clinical outcomes [14]. It is also important to highlight here that a study published in 2011 by Dakins et al. [28] stated that differences in QALYs might occur between treatment arms, even in cases where clinical outcomes are equal between intervention and usual care. We suggest that measures should be taken to improve the peer-review process for economic evaluations to avoid this kind of mistakes.

One feature of the transparency of results is the extent to which information related to the number of resources used has been properly reported in the economic evaluations. Only three studies [18,19,21] reported the number of resources used in physical units for all identified cost items. Most papers presented data on average cost or partially reported the number of resources, which made it difficult to assess the measurement of all previously identified costs in these articles.

Despite the challenges associated with the aging population, a holistic approach in terms of a societal perspective in EEs is preferred. A societal perspective includes indirect costs, such as productivity losses due to mortality and morbidity, costs and benefits to other societal sectors such as municipalities, forgone leisure time, time lost seeking medical services, and intangible costs in terms of pain or suffering. Since the studies included in this review focused on older adults, it is reasonable not to include labor market-related productivity losses. However, in most studies, other relevant indirect costs are not considered at all. For example, all evaluations that were assessed in this study focused on economic evaluations of mHealth interventions offered in a home-care setting. Therefore, it is reasonable to assume that these interventions had a positive impact on patients’ out of pocket expenses, simply because the patients could save on travel costs to hospitals. However, only one study included this cost [22]. Besides, although informal caregivers are essential in home-care settings, none of the studies discussed costs and benefits related to this aspect.

It is crucial to state the time-horizon explicitly in economic evaluations because it helps to recognize when costs and benefits are counted and the period over which costs should be spread. The time-horizon of most economic evaluations was of one year or less, which might not fully account for those benefits that are spread over more extended periods. This may imply an under-estimation of the cost-effectiveness of an intervention. Therefore, we suggest that longer time-horizons are useful in studies related to chronic conditions.

Sensitivity analyses are essential to test the robustness of results concerning changes in assumptions and the values of the input variables. However, only two studies [18,19] provided detailed justifications for their choice of parameters or ranges of parameter values in the analysis. Four other studies did not perform any sensitivity analysis [13,14,20,22].

### 4.1. Adherence to CHEERs List

The adherence to the CHEERs list can indicate the overall quality of reporting of a paper. We assigned scores generously, given that many items were partially reported in many studies. Four studies did not report source of funding [14,16,19,23] and two studies did not provide information describing the sources of their data on cost [13,17]. Out of 12 studies, only seven reported details on conducted sensitivity and uncertainty analyses, and most studies did not conduct a heterogeneity analysis. The majority of the studies failed to address the generalizability of their results to other settings [13,14,15,16,17,19,20,23]. 

Even though guidance for economic evaluations exists in terms of CHEERS checklist, most studies did not adhere to the standard guidelines. This precludes assessment of the quality of the studies. It is possible that the studies were performed in a rigorous manner, but the lack of appropriate reporting raises questions about their quality. One reason for the lack of information on the CHEERS checklist might be the word limit posed by the journals. We suggest that journal editors ask authors to include relevant information as a supplementary document to increase the quality of these studies. It should also be noted that the quality of reporting does not guarantee the quality of the study. Our findings regarding the lack of comprehensive reporting of recommended items and methodological rigor coincide with previous reviews [4,5].

### 4.2. Limitations

Our findings are based on the studies that were identified through the search strategy used for this review. Our search strategy may have missed important studies, although we find this unlikely. Another limitation is our subjective assessment of the quality of studies using the CHEERS list, which may result in disagreement about each study’s scores.

## 5. Conclusions

We found no evidence of cost-effectiveness for interventions related to complex smartphone communication, while we found some evidence for simple text-based communication-related interventions being cost-effective. Comprehensive economic evaluations of mHealth interventions designed for older adults over a more extended time period are warranted to be able to draw more robust conclusions.

## Figures and Tables

**Table 1:** Reported and evaluated cost-effectiveness of the identified mHealth interventions.

First Author, Year, Country	Effectiveness Measure	Reported	Evaluation	Remarks on Cost-Effectiveness
**Complex Smartphone Communication**				
Cubo E, 2016, Spain. [22]	UPDRS (I II III IV), QALY	Cost-effective in terms of UPDRS (II III IV)	Not reported if UPDRS (I II III IV), QALY and costs differ between alternatives.	Unknown due to lack of information
Gordon LG, 2014, Australia. [21]	QALY	dominant	Insignificant mean SF-6D score and mean annual total healthcare cost. Probabilistic sensitivity analysis showed 55.4% probability to be cost-effective at WTP threshold of AUS$50,000 (£33,000)/QALY	Not cost-effective
Stoddart A 2015, UK [18]	QALY	Not cost-effective	No significant differences in costs or QALY gain was observed for telemonitoring. CEAC showed 10.1% or 14.9% probability to be cost-effective at NICE threshold of £20,000 or £30,000, respectively.	Not cost-effective
Udsen FW, 2017, Denmark. [19]	QALY	Not cost-effective	No significant differences in costs or QALY gain was observed for tele-healthare. CEAC showed 50% probability of cost-effectiveness at €55,000 WTP for QALY.	Not cost-effective
Whittaker F, 2014, Australia. [14]	Cost savings	Cost-effective	Outcome was assumed to be equal between treatment alternatives. Not reported if costs differ between treatment alternatives.	Unknown due to lack of information
**Simple Text-Based Communication**				
Barnett T, 2007, USA. [13]	QALY	Cost-effective	Not reported if QALY and costs differ between alternatives.	Unknown due to lack of information
Burn E, 2017, Australia. [15]	QALY	Dominant	Significant differences were observed in costs and effects for Text me.	Cost-effective (Dominant)
Choi Yoo SJ, 2014, USA. [23]	DFD, QALY based on i)DFD, ii) SF-6D, and iii) modified EQ-5D	Cost-effective for DFD, QALY based on i) DFD, and ii) modified EQ-5D	Significant differences were observed in DFD, QALY based on (i) DFD, (ii) SF-6D and (iii) EQ-5D. Not reported if costs differ between alternatives.	Unknown due to lack of information
Cui Y, 2013, Canada. [16]	QALY	HLM not cost-effective	Simulation results showed that cost differences were not significant but QALY differences were significant for HL CEAC showed 95.4% probability to be cost-effective at $100,000 WTP for HL and 85.8 % probability to be cost-effective at threshold of CAD 50,000/QALY.	HLM not cost-effective
Katalenic B, 2015, USA. [20]	QALY	Cost-effective	QALY differences are not statistically or clinically significant (data not shown) Costs are significantly lower for DRMS.	Cost-effective
Maddison R, 2015, New Zealand. [17]	QALY, MET-hour of walking, leisure activity	Not cost-effective for QALY, cost-effective for both MET-hour of walking and leisure activity.	Significant differences were observed in MET-hour of walking and leisure activity in favor of the Heart intervention. No significant differences were observed in QALYs. Not reported if costs differ between alternatives. There would be a 72% and 90% probability of this intervention being cost-effective if WTP of the decision maker is NZD20,000 (€10,600) and NZD 50,000(€26,500).	Unknown due to lack of information for MET-hour of walking and leisure activity. Cost-effective for QALY based on threshold of USD 50 000 used in this study.

CEAC: cost-effectiveness acceptability curve; DFD: Depression free days; DRMS: diabetes remote monitoring and management system; HL: health lines; HLM: "Health Lines + Monitoring”; MET: metabolic equivalent; NICE: the national institute for health and care excellence; QALY: quality adjusted life years; UPDRS: unified Parkinson’s disease rating scale; WTP: willingness to pay.

**Table 2:** Decision rules for economic evaluations (intervention vs. comparator).

Scenarios	Cost	Outcome	Interpretation
1	↑	↑	Cost-effective if WTP exceeds the ICER
2	↓	↑	Cost-effective (intervention dominates the comparator)
3	≈	↑	Cost-effective (intervention dominates the comparator)
4	↑	↓	Not cost-effective (comparator dominates the intervention)
5	↓	↓	Cost-effective if willingness-to-accept exceeds the ICER
6	≈	↓	Not cost-effective (comparator dominates the intervention)
7	↑	≈	Not cost-effective (comparator dominates the intervention)
8	↓	≈	Cost-effective (intervention dominates the comparator i.e. cost-saving)
9	≈	≈	Not cost-effective (intervention and comparator are equal)

ICER: incremental cost-effectiveness ratio; WTP: willingness to pay. Abbreviation: ↑: statistically significantly higher; ↓: statistically significantly lower; ≈: no statistically significant differences Note: Reprinted from “Economic evaluation of interventions for the screening of Dementia”, by Saha, S., August 2018, Working paper 2018:20, Department of Economics, School of Economics and Management, Lund University.

## Supplementary Materials

The following are available online at https://www.mdpi.com/1660-4601/17/15/5290/s1, Table S1: Key words, Table S2: Characteristics of complex smartphone communication studies, Table S3: Characteristics of simple text-based communication studies, Table S4: Details of cost and effectiveness measure of complex smartphone communication studies, Table S5: Details of cost and effectiveness measure of simple text-based communication studies.
