# Supplementary material for: The Cost-Effectiveness of Mobile Health (mHealth) Interventions for Older Adults: Systematic Review
Source: Int J Environ Res Public Health. 2020 Jul 22;17(15):5290. doi: 10.3390/ijerph17155290 (PMC7432315; doi:10.3390/ijerph17155290)
Supplement: Supplementary file 1 [file ijerph-17-05290-s001.zip › Supplementary File 1 mHealth SLR 20191216-R1.docx]

**Supplementary File 1**

**Table S2.** Characteristics of complex smartphone communication studies.

| **First Author, Year, Country** | **Analysis; Study Design** | **Intervention** | **Comparator** | **Target Population; Sample Size;**  **Age** | **Perspective; Time Horizon** | **Price Year, Currency Used** | **Discount Rate** | **ICER/Results** | **Sensitivity Analysis** | **Source of Funding** | **CHEERS** |
| --- | --- | --- | --- | --- | --- | --- | --- | --- | --- | --- | --- |
| Cubo, 2016,  Spain. (22) | CEA,  Prospective, randomized, case-control study | home-based motor monitoring on a tablet software app, plus standard in-office visits | in-office visits | patients with advanced Parkinson’s disease (PD) with a Mini-Mental Scale11 score>24  total patients: 40  home based: 20  in-office: 20  home based: 66.44 years in-office: 66.05 years | Limited societal perspective (inferred)  One year | € | Not applicable | UPDRS-II ICER: € 325/ UPDRS  UPDRS-III ICER: €250/ UPDRS  UPDRS-IV ICER: €701/ UPDRS  UPDRS-total ICER: € 126.7/ UPDRS | Not reported | Industry | 21 |
| Gordon LG, 2014, Australia. (21) | CEA  Markov Model | TLC diabetes kit to monitor blood glucose. It contains a handbook, blood glucose meter, a box of test strips, a cell phone and a Bluetooth device | usual care | patients with established Type 2 diabetes mellitus and high glycated haemoglobin level.  starting age: 57 years | Healthcare perspective  5 years | 2011  Pounds  (AUD 1 = £0.657) | 5% | TLC dominant | univariate and PSA | Government | 23 |
| Stoddart A, 2015, UK. (18) | CUA,  RCT | Touch-screen telemonitoring equipment along with usual care | usual care | patients with COPD  total patients: 256  telemonitoring: 69.4 years control: 68.4 years | National Health Service perspective  12 months | 2010/11 financial year, £ | Not applicable | Base case (all admissions):  ICER, £ per QALY: 137 277 | change in baseline assumptions | Government | 22 |
| Udsen FW, 2017, Denmark. (19) | CEA; RCT | Telehealth care solution (a tablet with two apps and peripherals) in addition to usual care | usual care | patients with COPD with at least two exacerbations within the past 12 months.  total patients: 1225  telehealth: 578 patients  usual care: 647 patients  telehealth: 69.55 years  usual care: 70.33 years | healthcare and social sector perspective  12 months | 2014  Euro  (1€ = 7.4547 DKK) | Not applicable | ICER (adjusted, €per QALY): 55327 | PSA | Not reported | 22 |
| Whittaker F, 2014, Australia (14) | CBA, CEA,  RCT | home telehealth-based cardiac rehabilitation programme | standard hospital-based programme | CVD patients  total participants: 120  telehealth: 60 participants  usual care: 60 participants | provider’s perspective, and participant’s perspective,  6 months | Not reported | Not applicable |  | Not reported | Industry (inferred) | 15 |

CBA: cost benefit analysis; CEA: cost effectiveness analysis; COPD: chronic obstructive pulmonary disease; CUA: cost utility analysis; CVD: cardiovascular disease; GOLD: global initiative for obstructive lung disease; ICER: incremental cost effectiveness ratio; PSA: probabilistic sensitivity analysis; QALY: quality adjusted life years; RCT: randomized controlled trial; TLC: telephone-linked care; UPDRS: unified Parkinson’s disease rating scale functional status

**Table S3.** Characteristics of simple text-based communication studies.

| **First Author, Year, Country** | **Analysis; Study Design** | **Intervention** | **Comparator** | **Target Population; Sample Size;**  **Age** | **Perspective; Time Horizon** | **Price Year, Currency Used** | **Discount Rate** | **ICER** | **Sensitivity Analysis** | **Source of Funding** | **CHEERS** |
| --- | --- | --- | --- | --- | --- | --- | --- | --- | --- | --- | --- |
| Barnett,  2007,  USA. (13) | CUA,  Retrospective study | A cohort was observed after the introduction of the CCHT program for a period of 12 month.  CCHT includes messaging devices, videophones and disease management principles. Patients used the messaging device each day to answer questions about their diabetes symptoms and health status | A cohort was observed before the introduction of the CCHT program for a period of 12 month. | Veterans with diabetes if they had had two or more VA hospitalizations or VA emergency department visits in the 12 months prior to enrolment and were not institutionalized.  total participants: 370  68.2 years | Payer’s perspective (inferred);  12 months | Not reported | Not applicable | Mean ICER: $60,941/QALY | Not reported | Community Care Coordination Service. | 19 |
| Burn, 2017, Australia. (15) | DAM | Motivational text message to improve health-related behaviors plus usual care. | usual care | individuals with documented coronary heart disease (CHD)  total patients: 50 000  Male: 58 years  Female: 57 years | health system perspective;  lifetime | 2014 Australian dollars | 3% annually | Incremental cost per QALY: TEXT ME dominant | PSA  (one-way) and  scenario analyses. | Government and non-Governmental organizations | 23 |
| Choi Yoo SJ, 2014, USA. (23) | CEA,  Randomized trial | centralized telecare management coupled with automated symptom monitoring. | usual care | patients with cancer having pain (≥6 on the “worst pain in the past week” item of the Brief Pain Inventory (BPI) and depression (nine-item depression scale score≥10).  total participants: 405  intervention = 202;  usual care = 203  Mean Age: 58.8 years | payer's perspective;  12 months | USD | Not applicable | ICER:  DFD (complete follow-ups): US$ 19.72/DFD  DFD (complete and imputed follow ups): US$ 26.95/DFD  QALY (derived from DFD complete follow-up): US$ 18,017.73/QALY to US$ 36,035.45/QALY  QALY (derived from DFD complete and imputed follow-ups): US$ 24,774.38/QALY to US$ 49,548.75/QALY | poststart cost-effectiveness ratios were projected for new patients who might receive the 12-month intervention after the trial. | Not reported | 22 |
| Cui Y, 2013, Canada. (16) | CEA,  Randomized clinical trial | Group 2 received standard care plus Health Lines (HL): nurses were available on the telephone to provide suggestions about the patient’s daily management of the disease.  Group 3: "Health Lines + Monitoring (HL+M) group received the intervention plus an enhancement of in-home monitoring devices (weight scales and blood pressure monitors) and a computerized call schedule that prompted patients to enter in weights and blood pressures regularly throughout the year. | Group 1: standard care | patients with congestive heart failure  total participants: 179;  Average age: 75 years | healthcare system;  12 months | 2005 Canadian dollars | 0% | Standard care was strongly dominated by HL and HLM.  ICER for HL compared to HLM: CA$2 975/QALY | non-parametric bootstrap | Not reported | 21 |
| Katalenic B, 2015, USA. (20) | CEA, CMA,  Randomized controlled study | An automated Diabetes Remote Monitoring and Management System (DRMS) which used text messages or phone calls to remind patients to test their blood glucose and to report results. | standard care | patients with uncontrolled diabetes on insulin or patients with type 2 diabetes starting insulin.  total participants: 98  mean age, 59 years at the time of enrollment, | National health perspective (inferred);  6 months | Not reported | Not applicable | the DRMS intervention was cost-effective compared to usual care | Not reported | Government and Industry | 19 |
| Maddison R, 2015, New Zealand. (17) | CEA, CUA,  RCT | a mobile phone delivered intervention which includes automated package of text messages and a secure website with video messages aimed at increasing exercise behavior plus usual care | usual care alone | people with ischaemic heart disease (IHD) able to perform exercise and had access to the Internet.  HEART = 85  usual care = 86  Mean Age Intervention: 61.4 years control: 59.0 years | Healthcare (limited) (inferred);  24 weeks | 2012 $NZ  NZ$1 = €0.53 | Not applicable | ICER per QALY: NZ$28,768 (€15,247).  ICERs per MET-hour of walking and leisure activity: NZ$48 (€26) and NZ$74 ($€$39), respectively | change in baseline assumptions | Governmental organization | 21 |

CCHT: care coordination/home telehealth program; CEA: cost effectiveness analysis; CMA: cost minimization analysis; CUA: cost utility analysis; DAM: decision analytic model; DFD: Depression free days; ICER: incremental cost effectiveness ratio; PSA: probabilistic sensitivity analysis; QALY: quality adjusted life years; RCT: randomized controlled trial.

**Table S4.** Details of cost and effectiveness measure of complex smartphone communication studies.

| **First Author, Year** | **Type and Category of Costs** | **Data Source of Resource Use** | **Effectiveness Measure** | **Effectiveness Source** | **Methods of Valuation of Effects** |
| --- | --- | --- | --- | --- | --- |
| Cubo, 2016,  Spain. (22) | Direct medical costs  Pharmacological expenses  Non-medical costs,  the Kinesia systems (five devices for 20 patients) and delivery costs to the patients (€235 per patient). | Direct costs: standardized questionnaire, patient medical records and outside medical services information provided by patient/caregiver reports, which included medical visits, hospitalization, and goods and services used in the prevention, diagnosis, or treatment. Non-medical costs: patients and/or caregiver reports. | ICER-Unified Parkinson’s Disease Rating Scale (UPDRS)  ICER-QALY | UPDRS (I, II, III, IV subscales) rating scales and EQ-5D questionnaire were administered during each in-office visit. | preference scores for the  Spanish population is used for QALY. |
| Gordon 2014, Australia. (21) | Itemized labor and equipment cost (Bluetooth devices, cell phones and computer).  Healthcare cost (general practitioner and specialist visits, hospitalization and all medication use). | Prior study to collect mean cost of general classes of therapeutic drugs.  Hospitalization data from Queensland health admitted patients data collection.  Self-reports of other healthcare utilization. | QALY | SF-36 (SF-6D) | UK population scoring algorithm |
| Stoddart, 2015, UK. (18) | Direct cost (device cost, training cost),  Primary care medication associated with COPD. Secondary care costs (hospital admissions, emergency visits) | Direct cost of telemonitoring: private communication with NHS Lothian);  primary care and Accident and Emergency (A&E) visits**:** patient questionnaires at baseline and 12-month follow up, and by postal surveys at three-month intervals in-between. Telephone consultations: previously published surveys  Secondary care costs: patients’ secondary care records.  Study specific data: CRT teams recorded the length and nature of each contact with patients prospectively using timesheets. Practice nurse time spent training patients to use the equipment was estimated based on anecdotal descriptions from the staff who undertook the training multiplied by average hourly wage  Medication: British National Formulary pricing (under the curve approach) | QALY | The EQ-5D scores were measured at baseline and at one year. | The EQ-5D data collected are translated into ‘utility scores’ using the UK population tariff. QALYs were estimated using an area under the curve approach. |
| Udsen FW, 2017, Denmark. (19) | hospital services (in patient, out patient emergency), primary care, medicine, community care services (personal care, practical help, home nursing care and rehabilitation activities) ,  Intervention costs | healthcare and social care service use: register data  National patient-level data for all hospital contacts: Danish, National patient Register, contacts between patients and the primary care sector: National Health Insurance Service Register Medication: The Danish Register of Medicinal Product Statistics  Patient-level community care service use: individual care systems in each of the 26 included municipality districts. time registries in the municipality districts to keep record of time spent on monitoring  All hospital contacts: Danish Register for COPD. | Incremental costs per quality adjusted life-years gained from baseline | Information of mortalities: Danish Register of Causes of Death. Utility scores: EQ5D-3L health-related quality-of-life questionnaire at baseline. And at follow-up. | Utility scores: EQ5D-3L with Danish societal weights. QALYs were calculated by linear interpolation of utility scores. |
| Whittaker F, 2014, Australia. (14) | direct and indirect costs | Assessments, semi structured interviews, model consultations, financial reports and system outputs, Queensland Health and the Commonwealth Scientific and  Industrial Research Organisation (CSIRO), Finance Department of the  Prince Charles Hospital and the CSIRO. | Benefits | Assessments, semi structured interviews, model consultations, financial reports and system outputs. | Comparison of benefits was made on a per patient basis, using a cost/  benefits model developed by Whittaker. |

COPD: chronic obstructive pulmonary disease; CRT: the community respiratory team; ICER: incremental cost effectiveness ratio; NHS: national health service; QALY: quality adjusted life years.

**Table S5.** Details of cost and effectiveness measure of simple text-based communication studies.

| **First Author, Year** | **Type and Category of Costs** | **Data Source of Resource Use** | **Effectiveness Measure** | **Effectiveness Source** | **Methods of Valuation of Effects** |
| --- | --- | --- | --- | --- | --- |
| Barnett,  2007,  USA. (13) | direct costs to the Department of Veterans Affairs (VA) | Not reported | ICER  QALY | SF-36V questionnaire at baseline and at 12-month follow-up. | SF-36V is converted into a single health state utility score using the algorithm developed by Brazier et al. |
| Burn, 2017, Australia. (15) | primary care costs, prescriptions, hospital costs relating to major vascular events, intervention cost (database/IT infrastructure, project manager, clinical advisor, patient enrolment, messages, additional expenses) | Medicare Benefits Schedule and the Pharmaceutical benefit scheme,  healthcare expenditures on cardiovascular diseases  Consultation with the programme staff who used their experience and administrative records | QALY  MI avoided, Strokes avoided | RCT of Textme, where 710 participants were enrolled and previous studies | SF-12 survey was transformed to SF-6D using the algorithm given by Brazier et al.  QoL for MI ,CHD, history of stroke based on previous studies |
| Choi Yoo SJ, 2014, USA. (23) | intervention costs: physician, nurse care manager and automated monitoring set-up and maintenance costs | Intervention cost per patient was determined using provider payroll data and capital expenditure associated with the intervention.  Physician and nurse time cost was calculated based on administrative data on annual salary plus fringe and hours spent on the intervention during the year | depression-free days (DFD) and quality-adjusted life years. | Outcomes were assessed by blinded telephone interviews over 12 months (baseline and at Months 1, 3, 6 and 12, with some of the outcomes assessed less frequently). Depression, pain, mental health and disability outcomes were used to estimate the DFDs and QALYs associated with the intervention.  literature review | DFDs during the 12-month follow-up period were calculated from the HSCL-20 scores.  QALYs were derived from i)DFDs ii) SF-12 iii) modified EQ-5D survey iv) a visual analog scale. |
| Cui Y, 2013, Canada. (16) | direct costs. The intervention costs: all expenses from the healthcare sector associated with the program. Specific cost items included equipment and technology cost, personnel wages, technician assistance, travel expenses, administrative supports and supplies.  healthcare utilization costs:family physician visits, physician specialist visits, cardiac physician visits, internist specialist visits and hospital in-patient days. | Health Research Data Repository at the Manitoba Centre for Health Policy, University of Manitoba. | QALY | SF-36:Patient health outcome status surveys were conducted by mail, with follow-up over the phone to participants at baseline and at three, six and 12 months of the active intervention. | To obtain QALYs, a conversion formula developed by Brazier and colleagues (2002) was used to calculate the SF-6D utility score (QALYs) from SF-36 data. |
| Katalenic B, 2015, USA. (20) | setup and maintenance costs for the DRMS, telehealth services (a 20 min phone call).  Usual care: Standard visit costs, costs for follow-up phone calls.  Ideal care: physician visits and telephone consultation sessions | Medical Expenditure Panel Survey Data (MEPS), Medicare reimbursement rates, ATLANTUS trial  official statistics | QALY | Not reported | A validated prediction model of clinical characteristics was calculated both at the baseline time and in the post-test period. |
| Maddison R, 2015, New Zealand. (17) | costs of implementing and delivering the intervention only.  staff maintenance and web-hosting costs | Not reported | QALY | The EQ-5D (NZ tariff 2) was used to obtain a single preference index for the estimation of quality-adjusted life years (QALYs). | NZ tariff 2 |

CHD: Coronary Heart Disease; ICER: incremental cost effectiveness ratio; MI: myocardial infarction; QALY: quality adjusted life years; RCT: randomized controlled trial.
