# Supplementary material for: The Cost-Effectiveness of Mobile Health (mHealth) Interventions for Older Adults: Systematic Review
Source: Int J Environ Res Public Health. 2020 Jul 22;17(15):5290. doi: 10.3390/ijerph17155290 (PMC7432315; doi:10.3390/ijerph17155290)
Supplement: Supplementary file 1 [file ijerph-17-05290-s001.zip › Supplementary File 2 mHealth SLR 20191216-R1.docx]

**Supplementary File 2:**

**Table S1.** Key words.

| **Elderly** | | **Economic Evaluation** | | **Mobile or Computer Technology Intervention** | |
| --- | --- | --- | --- | --- | --- |
| ***Title/Abstract*** | ***MESH*** | ***Title/Abstract*** | ***MESH*** | ***Title/Abstract*** | ***MESH*** |
| "Aged" | "Aged" | "Health Impact Assessment" | "Health Impact Assessment" | "mobile health" | "Cell Phone" |
| "Middle Aged" | "Middle Aged" | "economic evaluation" | "Cost-Benefit Analysis" | "telehealth" | "Smartphone/ economics" |
| "elderly" |  | "cost benefit" |  | "telemedicine" | "Mobile Applications/ economics" |
|  |  | "cost-benefit" |  | "mhealth" | "Reminder Systems/economics" |
|  |  | "cost-utility" |  | "ehealth" | "Telemedicine/economics" |
|  |  | "cost utility" |  | "e-health" | "Telephone/economics" |
|  |  | "cost consequences" |  | "Cell Phone" | "Text Messaging/economics" |
|  |  | "cost-effectiveness" |  | "Telephone" |  |
|  |  | "cost-minimization" |  | "smart phone" |  |
|  |  | "cost effective" |  | "iphone" |  |
|  |  |  |  | "smartphone" |  |
|  |  |  |  | "Mobile Applications" |  |
|  |  |  |  | "mobile phone" |  |

**Search Strategies**

**Pubmed**

**search strategy April 22, 2018**

Middle Aged: 45-64 years

Aged: 65+ years

| [#6](https://www.ncbi.nlm.nih.gov/pubmed/advanced) | [Add](https://www.ncbi.nlm.nih.gov/pubmed/advanced) | Search **((((((("Aged"[Mesh])) OR "Middle Aged"[Mesh]) OR "Aged"[Title/Abstract]) OR "Middle Aged"[Title/Abstract]) OR "elderly"[Title/Abstract])) AND (((((((((((("Health Impact Assessment"[Mesh]) OR "Health Impact Assessment"[Title/Abstract]) OR "economic evaluation"[Title/Abstract]) OR "cost benefit"[Title/Abstract]) OR "cost-benefit"[Title/Abstract]) OR "cost-utility"[Title/Abstract]) OR "cost utility"[Title/Abstract]) OR "cost consequences"[Title/Abstract]) OR "cost-effectiveness"[Title/Abstract]) OR "cost-minimization"[Title/Abstract]) OR "cost effective"[Title/Abstract]) OR "Cost-Benefit Analysis"[Mesh])) AND (((((((((((((((((((("mobile health"[Title/Abstract]) OR "telehealth"[Title/Abstract]) OR "telemedicine"[Title/Abstract]) OR "mhealth"[Title/Abstract]) OR "ehealth"[Title/Abstract]) OR "e-health"[Title/Abstract]) OR "Cell Phone"[Mesh]) OR "Cell Phone"[Title/Abstract]) OR "Telephone"[Title/Abstract]) OR "smart phone"[Title/Abstract]) OR "iphone"[Title/Abstract]) OR "Smartphone/economics"[Mesh]) OR "smartphone"[Title/Abstract]) OR "Mobile Applications/economics"[Mesh]) OR "Mobile Applications"[Title/Abstract]) OR "mobile phone"[Title/Abstract]) OR "Reminder Systems/economics"[Mesh]) OR "Telemedicine/economics"[Mesh]) OR "Telephone/economics"[Mesh]) OR "Text Messaging/economics"[Mesh])** Sort by: **Best Match**Filters: **Publication date from 2007/01/01 to 2018/12/31; English** | [805](https://www.ncbi.nlm.nih.gov/pubmed/?cmd=HistorySearch&querykey=6) |
| --- | --- | --- | --- |
| [#5](https://www.ncbi.nlm.nih.gov/pubmed/advanced) | [Add](https://www.ncbi.nlm.nih.gov/pubmed/advanced) | Search **((((((("Aged"[Mesh])) OR "Middle Aged"[Mesh]) OR "Aged"[Title/Abstract]) OR "Middle Aged"[Title/Abstract]) OR "elderly"[Title/Abstract])) AND (((((((((((("Health Impact Assessment"[Mesh]) OR "Health Impact Assessment"[Title/Abstract]) OR "economic evaluation"[Title/Abstract]) OR "cost benefit"[Title/Abstract]) OR "cost-benefit"[Title/Abstract]) OR "cost-utility"[Title/Abstract]) OR "cost utility"[Title/Abstract]) OR "cost consequences"[Title/Abstract]) OR "cost-effectiveness"[Title/Abstract]) OR "cost-minimization"[Title/Abstract]) OR "cost effective"[Title/Abstract]) OR "Cost-Benefit Analysis"[Mesh])) AND (((((((((((((((((((("mobile health"[Title/Abstract]) OR "telehealth"[Title/Abstract]) OR "telemedicine"[Title/Abstract]) OR "mhealth"[Title/Abstract]) OR "ehealth"[Title/Abstract]) OR "e-health"[Title/Abstract]) OR "Cell Phone"[Mesh]) OR "Cell Phone"[Title/Abstract]) OR "Telephone"[Title/Abstract]) OR "smart phone"[Title/Abstract]) OR "iphone"[Title/Abstract]) OR "Smartphone/economics"[Mesh]) OR "smartphone"[Title/Abstract]) OR "Mobile Applications/economics"[Mesh]) OR "Mobile Applications"[Title/Abstract]) OR "mobile phone"[Title/Abstract]) OR "Reminder Systems/economics"[Mesh]) OR "Telemedicine/economics"[Mesh]) OR "Telephone/economics"[Mesh]) OR "Text Messaging/economics"[Mesh])** Sort by: **Best Match**Filters: **Publication date from 2007/01/01 to 2018/12/31** | [824](https://www.ncbi.nlm.nih.gov/pubmed/?cmd=HistorySearch&querykey=5) |
| [#4](https://www.ncbi.nlm.nih.gov/pubmed/advanced) | [Add](https://www.ncbi.nlm.nih.gov/pubmed/advanced) | Search **((((((("Aged"[Mesh])) OR "Middle Aged"[Mesh]) OR "Aged"[Title/Abstract]) OR "Middle Aged"[Title/Abstract]) OR "elderly"[Title/Abstract])) AND (((((((((((("Health Impact Assessment"[Mesh]) OR "Health Impact Assessment"[Title/Abstract]) OR "economic evaluation"[Title/Abstract]) OR "cost benefit"[Title/Abstract]) OR "cost-benefit"[Title/Abstract]) OR "cost-utility"[Title/Abstract]) OR "cost utility"[Title/Abstract]) OR "cost consequences"[Title/Abstract]) OR "cost-effectiveness"[Title/Abstract]) OR "cost-minimization"[Title/Abstract]) OR "cost effective"[Title/Abstract]) OR "Cost-Benefit Analysis"[Mesh])) AND (((((((((((((((((((("mobile health"[Title/Abstract]) OR "telehealth"[Title/Abstract]) OR "telemedicine"[Title/Abstract]) OR "mhealth"[Title/Abstract]) OR "ehealth"[Title/Abstract]) OR "e-health"[Title/Abstract]) OR "Cell Phone"[Mesh]) OR "Cell Phone"[Title/Abstract]) OR "Telephone"[Title/Abstract]) OR "smart phone"[Title/Abstract]) OR "iphone"[Title/Abstract]) OR "Smartphone/economics"[Mesh]) OR "smartphone"[Title/Abstract]) OR "Mobile Applications/economics"[Mesh]) OR "Mobile Applications"[Title/Abstract]) OR "mobile phone"[Title/Abstract]) OR "Reminder Systems/economics"[Mesh]) OR "Telemedicine/economics"[Mesh]) OR "Telephone/economics"[Mesh]) OR "Text Messaging/economics"[Mesh])** | [1120](https://www.ncbi.nlm.nih.gov/pubmed/?cmd=HistorySearch&querykey=4) |
| [#3](https://www.ncbi.nlm.nih.gov/pubmed/advanced) | [Add](https://www.ncbi.nlm.nih.gov/pubmed/advanced) | Search **((((((((((((((((((("mobile health"[Title/Abstract]) OR "telehealth"[Title/Abstract]) OR "telemedicine"[Title/Abstract]) OR "mhealth"[Title/Abstract]) OR "ehealth"[Title/Abstract]) OR "e-health"[Title/Abstract]) OR "Cell Phone"[Mesh]) OR "Cell Phone"[Title/Abstract]) OR "Telephone"[Title/Abstract]) OR "smart phone"[Title/Abstract]) OR "iphone"[Title/Abstract]) OR "Smartphone/economics"[Mesh]) OR "smartphone"[Title/Abstract]) OR "Mobile Applications/economics"[Mesh]) OR "Mobile Applications"[Title/Abstract]) OR "mobile phone"[Title/Abstract]) OR "Reminder Systems/economics"[Mesh]) OR "Telemedicine/economics"[Mesh]) OR "Telephone/economics"[Mesh]) OR "Text Messaging/economics"[Mesh]** | [82701](https://www.ncbi.nlm.nih.gov/pubmed/?cmd=HistorySearch&querykey=3) |
| [#2](https://www.ncbi.nlm.nih.gov/pubmed/advanced) | [Add](https://www.ncbi.nlm.nih.gov/pubmed/advanced) | Search **((((((((((("Health Impact Assessment"[Mesh]) OR "Health Impact Assessment"[Title/Abstract]) OR "economic evaluation"[Title/Abstract]) OR "cost benefit"[Title/Abstract]) OR "cost-benefit"[Title/Abstract]) OR "cost-utility"[Title/Abstract]) OR "cost utility"[Title/Abstract]) OR "cost consequences"[Title/Abstract]) OR "cost-effectiveness"[Title/Abstract]) OR "cost-minimization"[Title/Abstract]) OR "cost effective"[Title/Abstract]) OR "Cost-Benefit Analysis"[Mesh]** | [158418](https://www.ncbi.nlm.nih.gov/pubmed/?cmd=HistorySearch&querykey=2) |
| [#1](https://www.ncbi.nlm.nih.gov/pubmed/advanced) | [Add](https://www.ncbi.nlm.nih.gov/pubmed/advanced) | Search **(((("Aged"[Mesh])) OR "Middle Aged"[Mesh]) OR "Aged"[Title/Abstract]) OR "Middle Aged"[Title/Abstract]) OR "elderly"[Title/Abstract]** | [4856594](https://www.ncbi.nlm.nih.gov/pubmed/?cmd=HistorySearch&querykey=1) |

**Scopus April 22, 2018**

| 6 | ( ( TITLE-ABS-KEY ( aged )  OR  TITLE-ABS-KEY ( {Middle Aged} )  OR  TITLE-ABS-KEY ( elderly ) ) )  AND  ( ( TITLE-ABS-KEY ( {Health Impact Assessment} )  OR  TITLE-ABS-KEY ( {economic evaluation} )  OR  TITLE-ABS-KEY ( "cost benefit" )  OR  TITLE-ABS-KEY ( "cost-utility" )  OR  TITLE-ABS-KEY ( "cost consequences" )  OR  TITLE-ABS-KEY ( "cost-minimization" )  OR  TITLE-ABS-KEY ( "cost effective" )  OR  TITLE-ABS-KEY ( "Cost-Benefit Analysis" ) ) )  AND  ( ( TITLE-ABS-KEY ( {mobile health} )  OR  TITLE-ABS-KEY ( "telehealth" )  OR  TITLE-ABS-KEY ( "telemedicine" )  OR  TITLE-ABS-KEY ( "mhealth" )  OR  TITLE-ABS-KEY ( "ehealth" )  OR  TITLE-ABS-KEY ( "Cell Phone" )  OR  TITLE-ABS-KEY ( "Telephone" )  OR  TITLE-ABS-KEY ( {smart phone} )  OR  TITLE-ABS-KEY ( {iphone} )  OR  TITLE-ABS-KEY ( "Mobile Applications" )  OR  TITLE-ABS-KEY ( {mobile phone} )  OR  TITLE-ABS-KEY ( {Reminder Systems} )  OR  TITLE-ABS-KEY ( {Text Messaging} ) ) )  AND  ( LIMIT-TO ( LANGUAGE ,  "English" ) )  AND  ( LIMIT-TO ( PUBYEAR ,  2018 )  OR  LIMIT-TO ( PUBYEAR ,  2017 )  OR  LIMIT-TO ( PUBYEAR ,  2016 )  OR  LIMIT-TO ( PUBYEAR ,  2015 )  OR  LIMIT-TO ( PUBYEAR ,  2014 )  OR  LIMIT-TO ( PUBYEAR ,  2013 )  OR  LIMIT-TO ( PUBYEAR ,  2012 )  OR  LIMIT-TO ( PUBYEAR ,  2011 )  OR  LIMIT-TO ( PUBYEAR ,  2010 )  OR  LIMIT-TO ( PUBYEAR ,  2009 )  OR  LIMIT-TO ( PUBYEAR ,  2008 )  OR  LIMIT-TO ( PUBYEAR ,  2007 ) ) View More | [946 document results](https://www-scopus-com.miman.bib.bth.se/search/history/results.uri?origin=searchhistory&shid=6) |
| --- | --- | --- |
| 5 | ( ( TITLE-ABS-KEY ( aged )  OR  TITLE-ABS-KEY ( {Middle Aged} )  OR  TITLE-ABS-KEY ( elderly ) ) )  AND  ( ( TITLE-ABS-KEY ( {Health Impact Assessment} )  OR  TITLE-ABS-KEY ( {economic evaluation} )  OR  TITLE-ABS-KEY ( "cost benefit" )  OR  TITLE-ABS-KEY ( "cost-utility" )  OR  TITLE-ABS-KEY ( "cost consequences" )  OR  TITLE-ABS-KEY ( "cost-minimization" )  OR  TITLE-ABS-KEY ( "cost effective" )  OR  TITLE-ABS-KEY ( "Cost-Benefit Analysis" ) ) )  AND  ( ( TITLE-ABS-KEY ( {mobile health} )  OR  TITLE-ABS-KEY ( "telehealth" )  OR  TITLE-ABS-KEY ( "telemedicine" )  OR  TITLE-ABS-KEY ( "mhealth" )  OR  TITLE-ABS-KEY ( "ehealth" )  OR  TITLE-ABS-KEY ( "Cell Phone" )  OR  TITLE-ABS-KEY ( "Telephone" )  OR  TITLE-ABS-KEY ( {smart phone} )  OR  TITLE-ABS-KEY ( {iphone} )  OR  TITLE-ABS-KEY ( "Mobile Applications" )  OR  TITLE-ABS-KEY ( {mobile phone} )  OR  TITLE-ABS-KEY ( {Reminder Systems} )  OR  TITLE-ABS-KEY ( {Text Messaging} ) ) )  AND  ( LIMIT-TO ( LANGUAGE ,  "English" ) ) View More | [1,324 document results](https://www-scopus-com.miman.bib.bth.se/search/history/results.uri?origin=searchhistory&shid=5) |

| ( ( TITLE-ABS-KEY ( aged )  OR  TITLE-ABS-KEY ( {Middle Aged} )  OR  TITLE-ABS-KEY ( elderly ) ) )  AND  ( ( TITLE-ABS-KEY ( {Health Impact Assessment} )  OR  TITLE-ABS-KEY ( {economic evaluation} )  OR  TITLE-ABS-KEY ( "cost benefit" )  OR  TITLE-ABS-KEY ( "cost-utility" )  OR  TITLE-ABS-KEY ( "cost consequences" )  OR  TITLE-ABS-KEY ( "cost-minimization" )  OR  TITLE-ABS-KEY ( "cost effective" )  OR  TITLE-ABS-KEY ( "Cost-Benefit Analysis" ) ) )  AND  ( ( TITLE-ABS-KEY ( {mobile health} )  OR  TITLE-ABS-KEY ( "telehealth" )  OR  TITLE-ABS-KEY ( "telemedicine" )  OR  TITLE-ABS-KEY ( "mhealth" )  OR  TITLE-ABS-KEY ( "ehealth" )  OR  TITLE-ABS-KEY ( "Cell Phone" )  OR  TITLE-ABS-KEY ( "Telephone" )  OR  TITLE-ABS-KEY ( {smart phone} )  OR  TITLE-ABS-KEY ( {iphone} )  OR  TITLE-ABS-KEY ( "Mobile Applications" )  OR  TITLE-ABS-KEY ( {mobile phone} )  OR  TITLE-ABS-KEY ( {Reminder Systems} )  OR  TITLE-ABS-KEY ( {Text Messaging} ) ) ) View More | [1,358 document results](https://www-scopus-com.miman.bib.bth.se/search/history/results.uri?origin=searchhistory&shid=4) |
| --- | --- |

| 3 | ( TITLE-ABS-KEY ( {mobile health} )  OR  TITLE-ABS-KEY ( "telehealth" )  OR  TITLE-ABS-KEY ( "telemedicine" )  OR  TITLE-ABS-KEY ( "mhealth" )  OR  TITLE-ABS-KEY ( "ehealth" )  OR  TITLE-ABS-KEY ( "Cell Phone" )  OR  TITLE-ABS-KEY ( "Telephone" )  OR  TITLE-ABS-KEY ( {smart phone} )  OR  TITLE-ABS-KEY ( {iphone} )  OR  TITLE-ABS-KEY ( "Mobile Applications" )  OR  TITLE-ABS-KEY ( {mobile phone} )  OR  TITLE-ABS-KEY ( {Reminder Systems} )  OR  TITLE-ABS-KEY ( {Text Messaging} ) ) View More | [245,443 document results](https://www-scopus-com.miman.bib.bth.se/search/history/results.uri?origin=searchhistory&shid=3) |
| --- | --- | --- |

| 2 | ( TITLE-ABS-KEY ( {Health Impact Assessment} )  OR  TITLE-ABS-KEY ( {economic evaluation} )  OR  TITLE-ABS-KEY ( "cost benefit" )  OR  TITLE-ABS-KEY ( "cost-utility" )  OR  TITLE-ABS-KEY ( "cost consequences" )  OR  TITLE-ABS-KEY ( "cost-minimization" )  OR  TITLE-ABS-KEY ( "cost effective" )  OR  TITLE-ABS-KEY ( "Cost-Benefit Analysis" ) ) | [384,929 document results](https://www-scopus-com.miman.bib.bth.se/search/history/results.uri?origin=searchhistory&shid=2) |  |
| --- | --- | --- | --- |

| 1 | ( TITLE-ABS-KEY ( aged )  OR  TITLE-ABS-KEY ( {Middle Aged} )  OR  TITLE-ABS-KEY ( elderly ) ) | | [4,731,330 document results](https://www-scopus-com.miman.bib.bth.se/search/history/results.uri?origin=searchhistory&shid=1) | |  |
| --- | --- | --- | --- | --- | --- |
|  |  | |  | |  |

**CINAHL April 22, 2018**

| S16 | S11 AND S12 AND S13 | **Limiters** - Published Date: 20070101-20181231; Language: English  **Search modes** - Boolean/Phrase | [**View Results**](javascript:__doPostBack('ctl00$ctl00$FindField$FindField$historyControl$HistoryRepeater$ctl00$linkResults','')) (102)  [**View Details**](javascript:showShDetails(%22ctl00_ctl00_FindField_FindField_historyControl_ctrlPopup%22,%20%22S16%22);)  [**Edit**](http://web.a.ebscohost.com.miman.bib.bth.se/Legacy/Views/UserControls/Ehost/) |
| --- | --- | --- | --- |

| S14 | S11 AND S12 AND S13 | **Search modes** - Boolean/Phrase | [**View Results**](javascript:__doPostBack('ctl00$ctl00$FindField$FindField$historyControl$HistoryRepeater$ctl00$linkResults','')) (126)  [**View Details**](javascript:showShDetails(%22ctl00_ctl00_FindField_FindField_historyControl_ctrlPopup%22,%20%22S14%22);)  [**Edit**](http://web.a.ebscohost.com.miman.bib.bth.se/Legacy/Views/UserControls/Ehost/) |  |
| --- | --- | --- | --- | --- |
|  | S13 | S6 OR S7 OR S8 OR S9 OR S10 | **Search modes** - Boolean/Phrase | [**View Results**](javascript:__doPostBack('ctl00$ctl00$FindField$FindField$historyControl$HistoryRepeater$ctl01$linkResults','')) (47,523)  [**View Details**](javascript:showShDetails(%22ctl00_ctl00_FindField_FindField_historyControl_ctrlPopup%22,%20%22S13%22);)  [**Edit**](http://web.a.ebscohost.com.miman.bib.bth.se/Legacy/Views/UserControls/Ehost/) |
|  | S12 | S3 OR S4 OR S5 | **Search modes** - Boolean/Phrase | [**View Results**](javascript:__doPostBack('ctl00$ctl00$FindField$FindField$historyControl$HistoryRepeater$ctl02$linkResults','')) (36,876)  [**View Details**](javascript:showShDetails(%22ctl00_ctl00_FindField_FindField_historyControl_ctrlPopup%22,%20%22S12%22);)  [**Edit**](http://web.a.ebscohost.com.miman.bib.bth.se/Legacy/Views/UserControls/Ehost/) |
|  | S11 | S1 OR S2 | **Search modes** - Boolean/Phrase | [**View Results**](javascript:__doPostBack('ctl00$ctl00$FindField$FindField$historyControl$HistoryRepeater$ctl03$linkResults','')) (197,640)  [**View Details**](javascript:showShDetails(%22ctl00_ctl00_FindField_FindField_historyControl_ctrlPopup%22,%20%22S11%22);)  [**Edit**](http://web.a.ebscohost.com.miman.bib.bth.se/Legacy/Views/UserControls/Ehost/) |
|  | S10 | (MM "Telehealth+") OR (MM "Telemedicine+") OR (MM "Cellular Phone+") OR (MM "Telephone+") OR (MM "Smartphone+") OR (MM "Text Messaging") OR (MM "Mobile Applications") OR (MM "Reminder Systems") | **Search modes** - Boolean/Phrase | [**View Results**](javascript:__doPostBack('ctl00$ctl00$FindField$FindField$historyControl$HistoryRepeater$ctl04$linkResults','')) (20,173)  [**View Details**](javascript:showShDetails(%22ctl00_ctl00_FindField_FindField_historyControl_ctrlPopup%22,%20%22S10%22);)  [**Edit**](http://web.a.ebscohost.com.miman.bib.bth.se/Legacy/Views/UserControls/Ehost/) |
|  | S9 | AB "mobile phone" OR AB "Reminder Systems" OR AB "Text Messaging" | **Search modes** - Boolean/Phrase | [**View Results**](javascript:__doPostBack('ctl00$ctl00$FindField$FindField$historyControl$HistoryRepeater$ctl05$linkResults','')) (1,924)  [**View Details**](javascript:showShDetails(%22ctl00_ctl00_FindField_FindField_historyControl_ctrlPopup%22,%20%22S9%22);)  [**Edit**](http://web.a.ebscohost.com.miman.bib.bth.se/Legacy/Views/UserControls/Ehost/) |
|  | S8 | TI "mobile phone" OR TI "Reminder Systems" OR TI "Text Messaging" | **Search modes** - Boolean/Phrase | [**View Results**](javascript:__doPostBack('ctl00$ctl00$FindField$FindField$historyControl$HistoryRepeater$ctl06$linkResults','')) (1,217)  [**View Details**](javascript:showShDetails(%22ctl00_ctl00_FindField_FindField_historyControl_ctrlPopup%22,%20%22S8%22);)  [**Edit**](http://web.a.ebscohost.com.miman.bib.bth.se/Legacy/Views/UserControls/Ehost/) |
|  | S7 | AB "mobile health" OR AB "telehealth" OR AB "telemedicine" OR AB "mhealth" OR AB "ehealth" OR AB "e-health" OR AB "Cell Phone" OR AB "Telephone" OR AB "smart phone" OR AB iphone OR AB "smartphone" OR AB "Mobile Applications" | **Search modes** - Boolean/Phrase | [**View Results**](javascript:__doPostBack('ctl00$ctl00$FindField$FindField$historyControl$HistoryRepeater$ctl07$linkResults','')) (28,717)  [**View Details**](javascript:showShDetails(%22ctl00_ctl00_FindField_FindField_historyControl_ctrlPopup%22,%20%22S7%22);)  [**Edit**](http://web.a.ebscohost.com.miman.bib.bth.se/Legacy/Views/UserControls/Ehost/) |
|  | S6 | TI "mobile health" OR TI "telehealth" OR TI "telemedicine" OR TI "mhealth" OR TI "ehealth" OR TI "e-health" OR TI "Cell Phone" OR TI "Telephone" OR TI "smart phone" OR TI iphone OR TI "smartphone" OR TI "Mobile Applications" | **Search modes** - Boolean/Phrase | [**View Results**](javascript:__doPostBack('ctl00$ctl00$FindField$FindField$historyControl$HistoryRepeater$ctl08$linkResults','')) (12,263)  [**View Details**](javascript:showShDetails(%22ctl00_ctl00_FindField_FindField_historyControl_ctrlPopup%22,%20%22S6%22);)  [**Edit**](http://web.a.ebscohost.com.miman.bib.bth.se/Legacy/Views/UserControls/Ehost/) |
|  | S5 | (MM "Health Impact Assessment") OR (MM "Cost Benefit Analysis") | **Search modes** - Boolean/Phrase | [**View Results**](javascript:__doPostBack('ctl00$ctl00$FindField$FindField$historyControl$HistoryRepeater$ctl09$linkResults','')) (5,718)  [**View Details**](javascript:showShDetails(%22ctl00_ctl00_FindField_FindField_historyControl_ctrlPopup%22,%20%22S5%22);)  [**Edit**](http://web.a.ebscohost.com.miman.bib.bth.se/Legacy/Views/UserControls/Ehost/) |
|  | S4 | AB "Health Impact Assessment" OR AB "economic evaluation" OR AB "cost benefit" OR AB "cost-benefit" OR AB "cost-utility" OR AB "cost utility" OR AB "cost consequences" OR AB "cost-effectiveness" OR AB "cost-minimization" OR AB "cost effective" OR AB "Cost-Benefit Analysis" | **Search modes** - Boolean/Phrase | [**View Results**](javascript:__doPostBack('ctl00$ctl00$FindField$FindField$historyControl$HistoryRepeater$ctl10$linkResults','')) (27,774)  [**View Details**](javascript:showShDetails(%22ctl00_ctl00_FindField_FindField_historyControl_ctrlPopup%22,%20%22S4%22);)  [**Edit**](http://web.a.ebscohost.com.miman.bib.bth.se/Legacy/Views/UserControls/Ehost/) |
|  | S3 | TI "Health Impact Assessment" OR TI "economic evaluation" OR TI "cost benefit" OR TI "cost-benefit" OR TI "cost-utility" OR TI "cost utility" OR TI "cost consequences" OR TI "cost-effectiveness" OR TI "cost-minimization" OR TI "cost effective" OR TI "Cost-Benefit Analysis" | **Search modes** - Boolean/Phrase | [**View Results**](javascript:__doPostBack('ctl00$ctl00$FindField$FindField$historyControl$HistoryRepeater$ctl11$linkResults','')) (13,483)  [**View Details**](javascript:showShDetails(%22ctl00_ctl00_FindField_FindField_historyControl_ctrlPopup%22,%20%22S3%22);)  [**Edit**](http://web.a.ebscohost.com.miman.bib.bth.se/Legacy/Views/UserControls/Ehost/) |
|  | S2 | (MM "Aged+") OR (MM "Middle Age") | **Search modes** - Boolean/Phrase | [**View Results**](javascript:__doPostBack('ctl00$ctl00$FindField$FindField$historyControl$HistoryRepeater$ctl12$linkResults','')) (9,830)  [**View Details**](javascript:showShDetails(%22ctl00_ctl00_FindField_FindField_historyControl_ctrlPopup%22,%20%22S2%22);)  [**Edit**](http://web.a.ebscohost.com.miman.bib.bth.se/Legacy/Views/UserControls/Ehost/) |
|  | S1 | TI "Aged" OR TI "Middle Aged" OR TI "elderly" OR AB "Aged" OR AB "Middle Aged" OR AB "elderly" | **Search modes** - Boolean/Phrase | [**View Results**](javascript:__doPostBack('ctl00$ctl00$FindField$FindField$historyControl$HistoryRepeater$ctl13$linkResults','')) (192,030) |
